# Supplementary material for: HURP regulates Kif18A recruitment and activity to synergistically control microtubule dynamics
Source: Nat Commun. 2024 Nov 8;15:9687. doi: 10.1038/s41467-024-53691-7 (PMC11549086; doi:10.1038/s41467-024-53691-7)
Supplement: Supplementary file 1 — Supplementary Information [file 41467_2024_53691_MOESM1_ESM.pdf]

HURP regulates Kif18A recruitment and activity to synergistically control microtubule dynamics

Supplementary Information

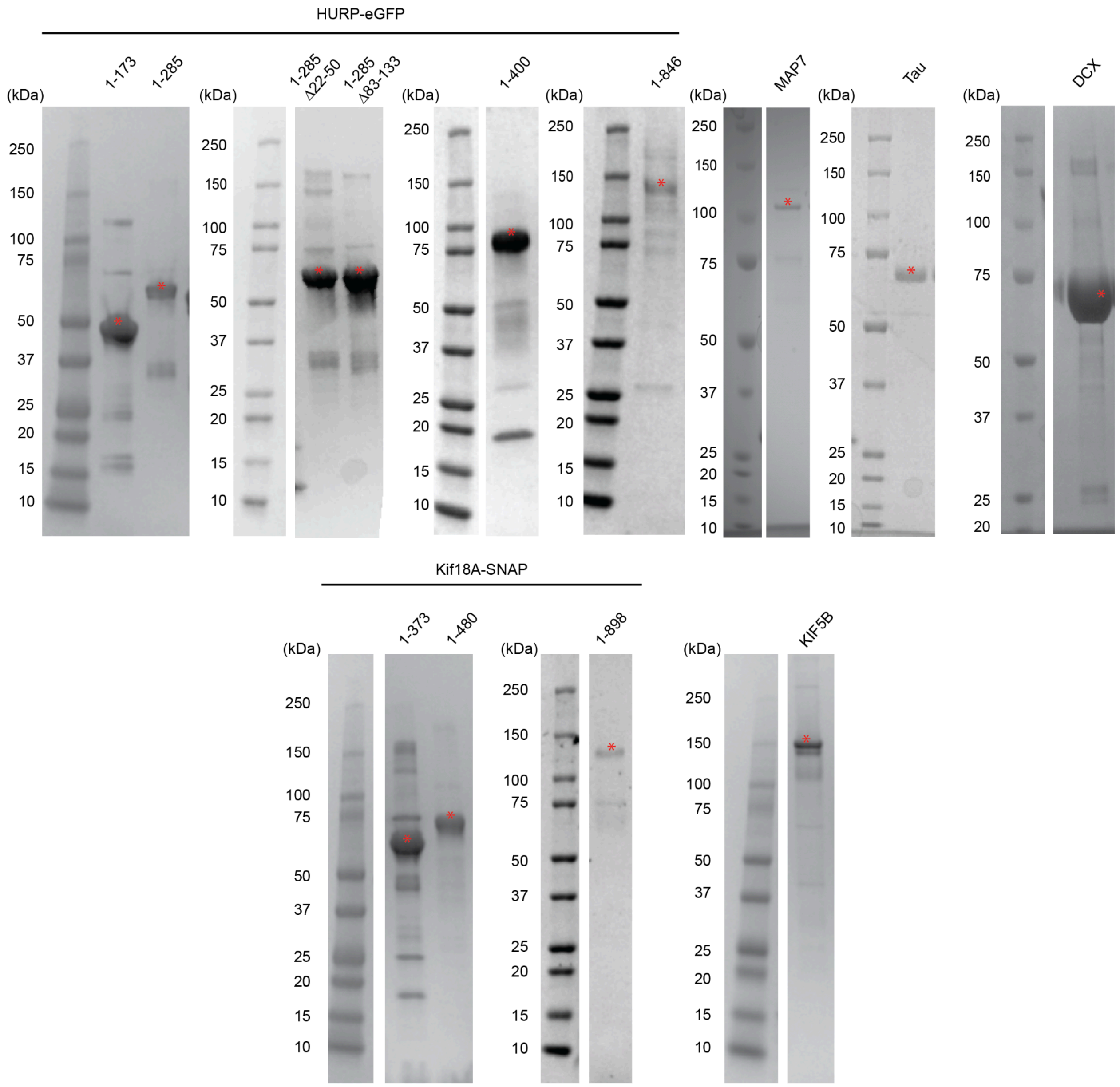

Supplementary Figure 1. SDS-PAGE analysis of protein constructs. Coomassie blue staining of 4-20% polyacrylamide gels showing all constructs used in this work. The band corresponding to the protein of interest is marked with a red asterisk.

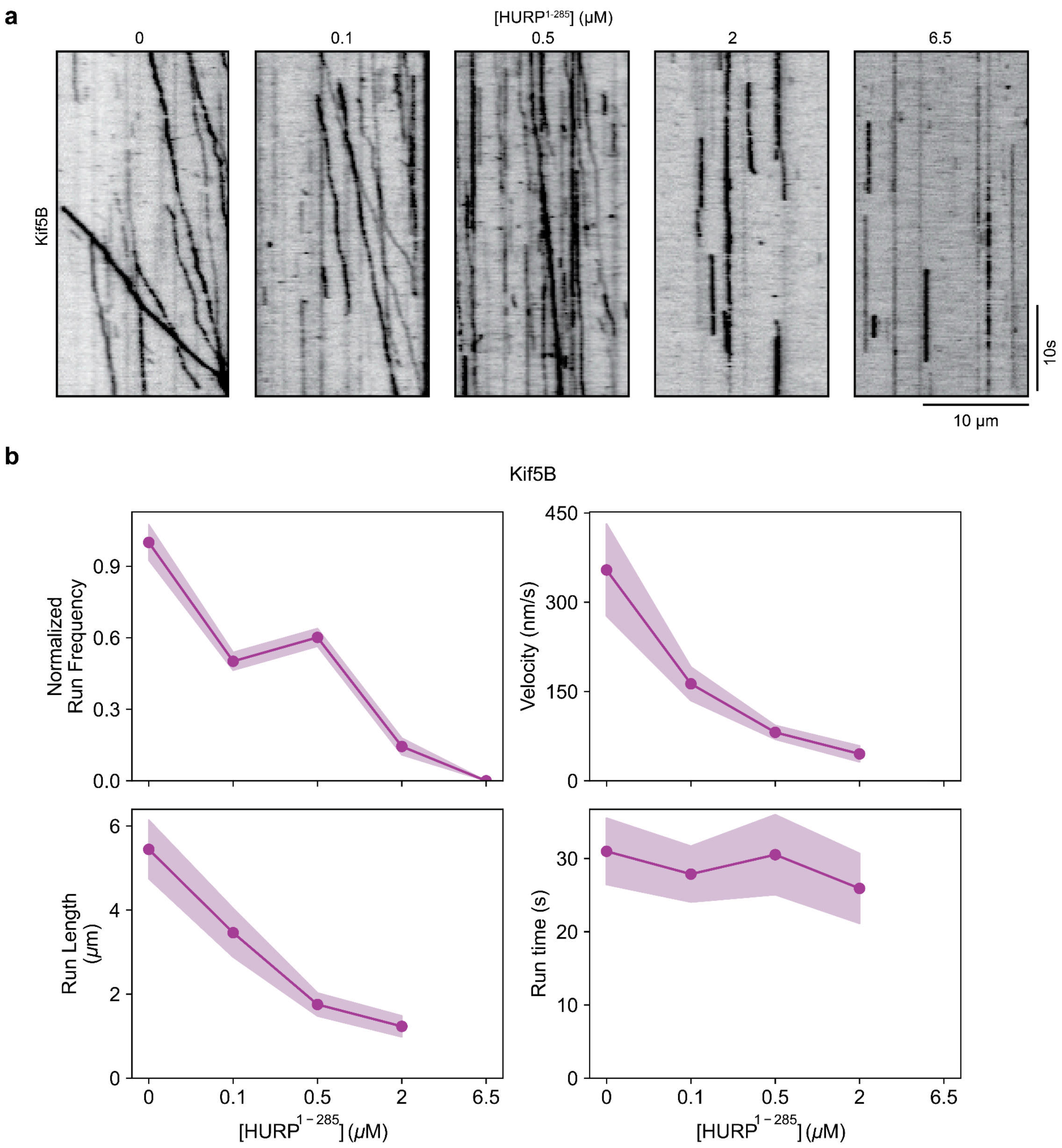

Supplementary Figure 2. HURP fails to activate Kif5B motility. **a**. Representative kymographs showing the motility of full-length Kif5B in the presence of increasing HURP$^{1\text{-}285}$. **b**. Normalized run frequency (N = 10 kymographs for each condition), velocity, run length and run time (N = 25 motors for each condition) of Kif5B for different HURP$^{1\text{-}285}$ concentrations. The line and shadows represent the mean and S.E., respectively. Source data are provided as a Source Data file.

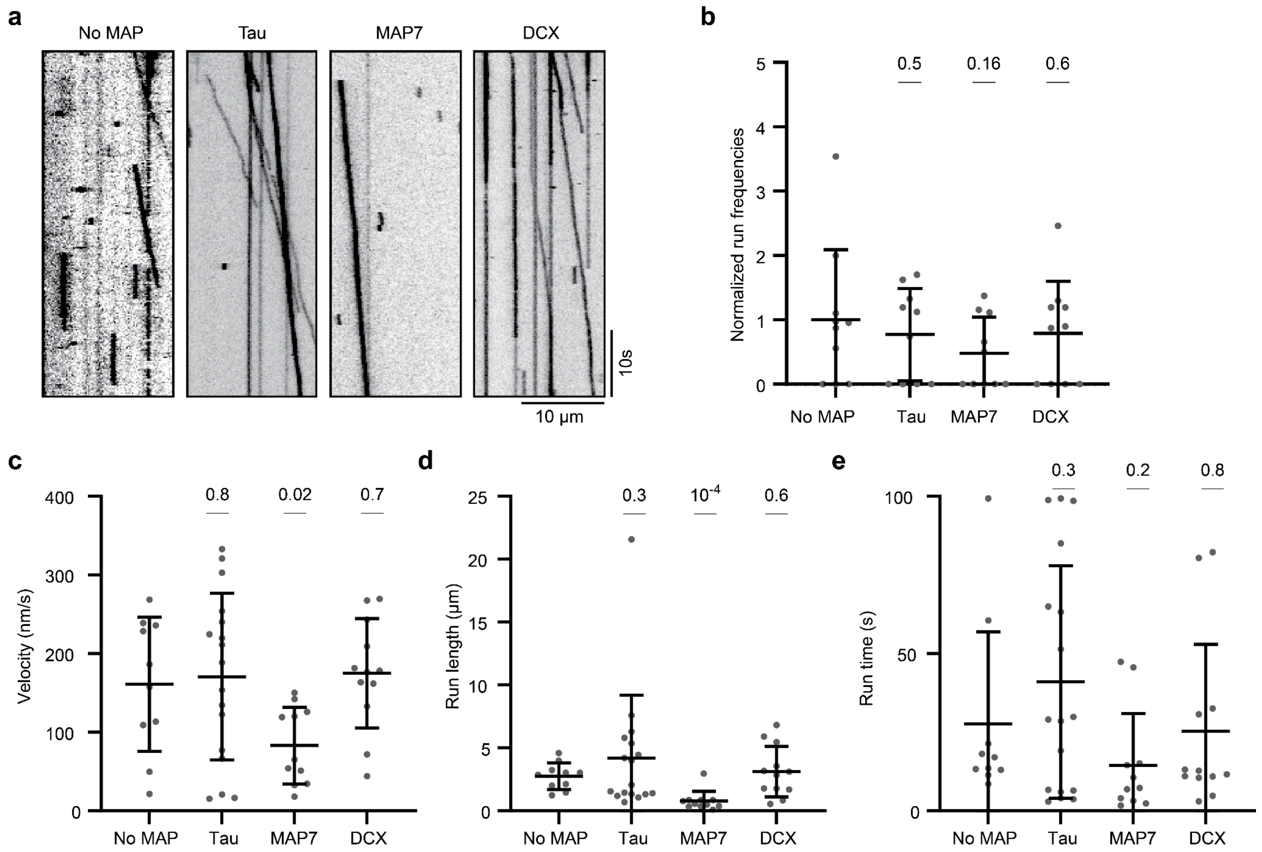

Supplementary Figure 3. Tau, MAP7 and DCX fail to activate Kif18A motility. a. Representative kymographs showing motility of Kif18A in the presence of tau, MAP7 and DCX. **b - e.** Normalized run frequency (**b**) , velocity (**c**), run length (**d**) and run time (**e**) of Kif18A in the presence of tau, MAP7 or DCX (For **b**, N = 10 kymographs for each condition; for **c** - **e**, N = 10, 17, 11, 12 motors). The center line and whiskers represent the mean and S.D., respectively. P values are calculated from a two-tailed t-test. Source data are provided as a Source Data file.

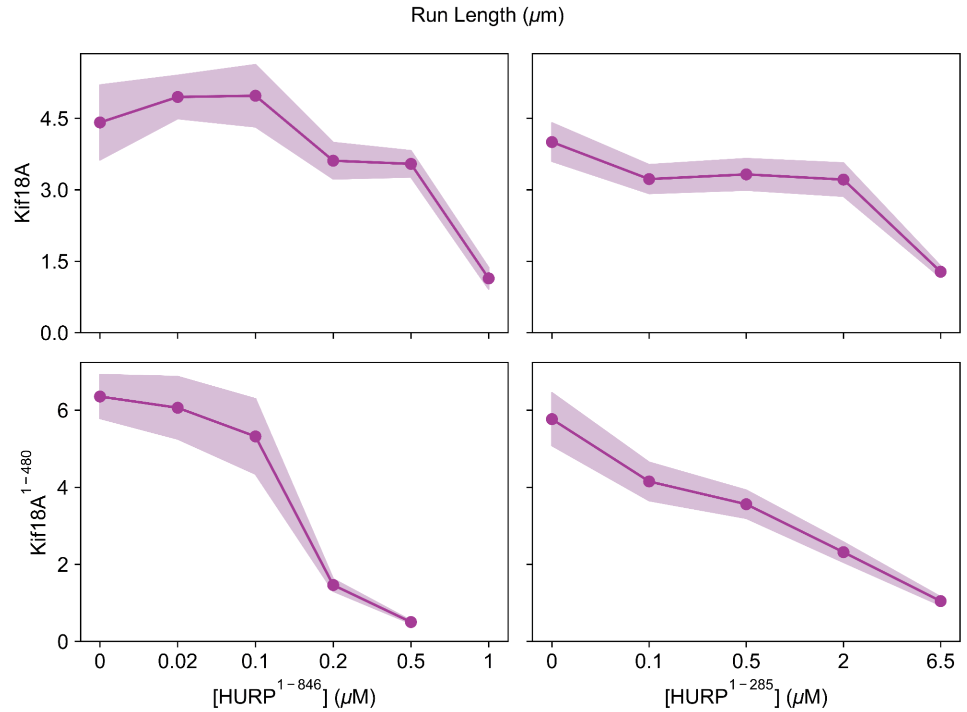

Supplementary Figure 4. High concentrations of HURP restrict the run length of Kif18A. Run length of Kif18A with titrated HURP (upper left, N = 32, 50, 33, 48, 50, 40 motors, respectively), Kif18A with titrated HURP$^{1\text{-}285}$ (upper right, N = 51, 44, 54, 52, 52 motors, respectively), Kif18A$^{1\text{-}480}$ with titrated HURP (lower left, N = 25 motor for each data point) and Kif18A$^{1\text{-}480}$ with titrated HURP$^{1\text{-}285}$ (lower right, N = 52, 51, 52, 52, 52 motors, respectively). The line and shadows represent the mean and S.E., respectively. Source data are provided as a Source Data file.

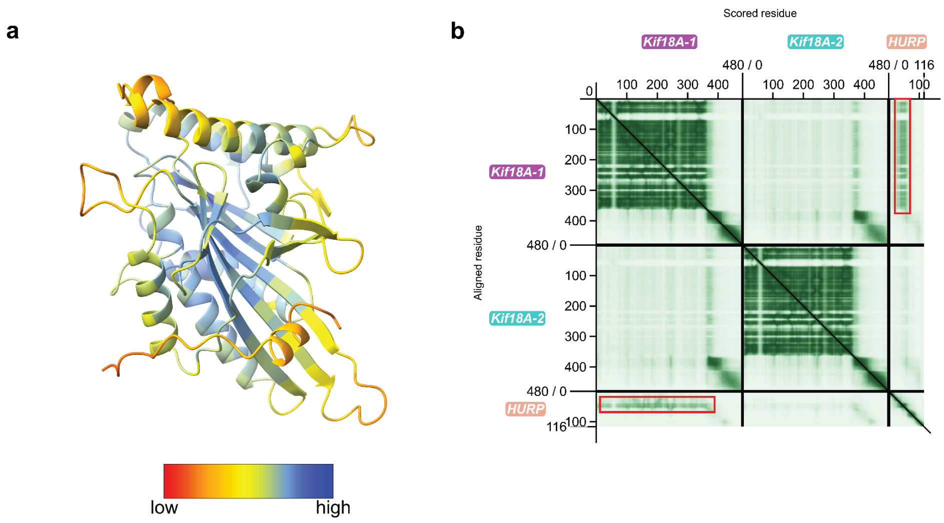

Supplementary Figure 5. Confidence metrics for AlphaFold 3 predictions. a. Representative Kif18A-HURP predicted model colored by plDDT score (only one Kif18A motor region and HURP interacting motif are shown for simplicity). **b.** Predicted aligned error (PAE) matrix for the corresponding predicted model. Red rectangles highlight the regions in the model shown in **a.** Greener values in quadrants associated to different subunits generally imply higher confidence in the interaction between those subunits. PAE matrices for the other models are similar and are omitted for simplicity.

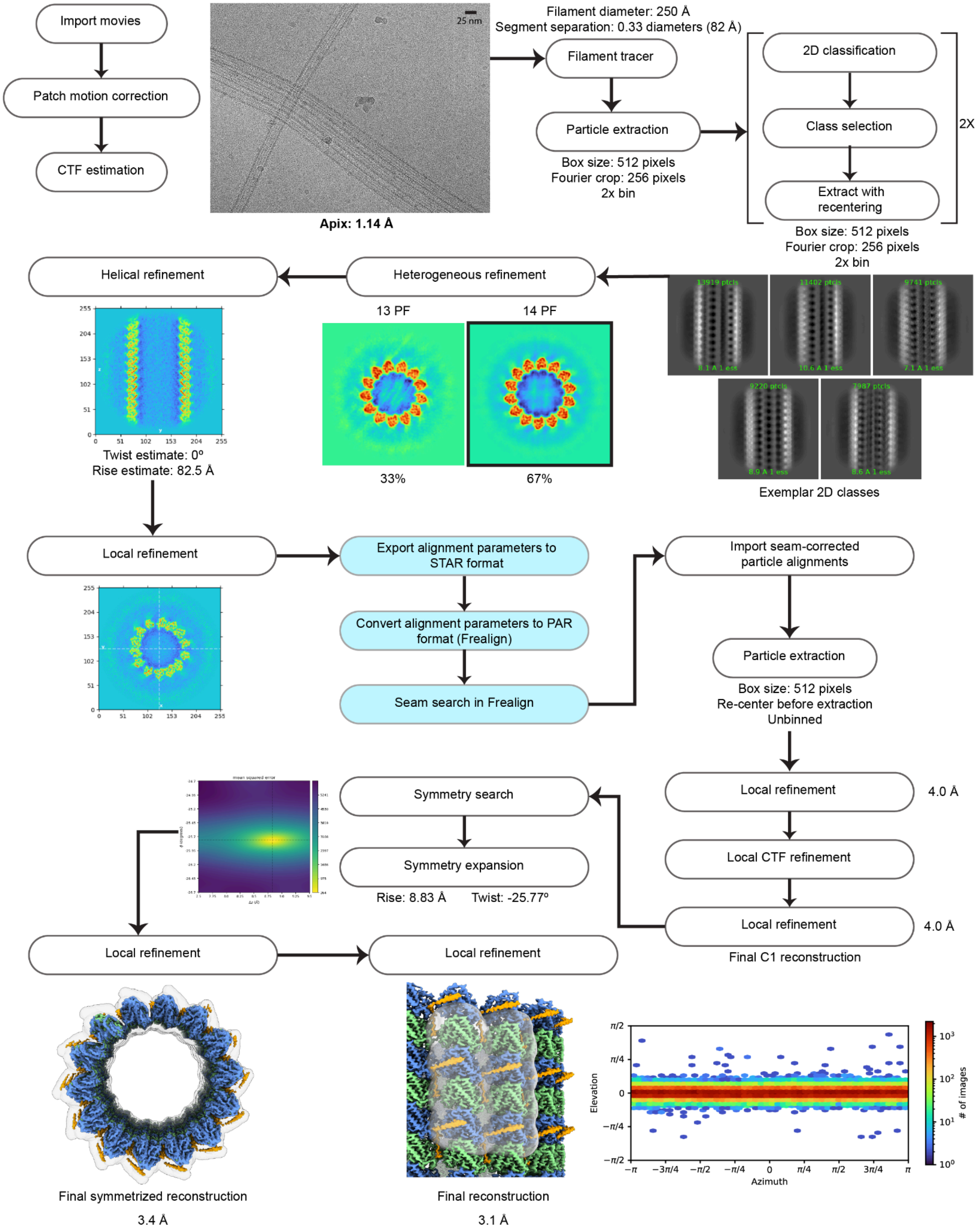

Supplementary Figure 6. Cryo-EM data processing for the microtubule-HURP dataset. Processing pipeline applied to the HURP-bound microtubule dataset. Unless specified otherwise, all

steps were performed in CryoSparc. Boxes in light blue include steps implemented outside of the CryoSparc software package. Masks are shown with transparent surfaces.

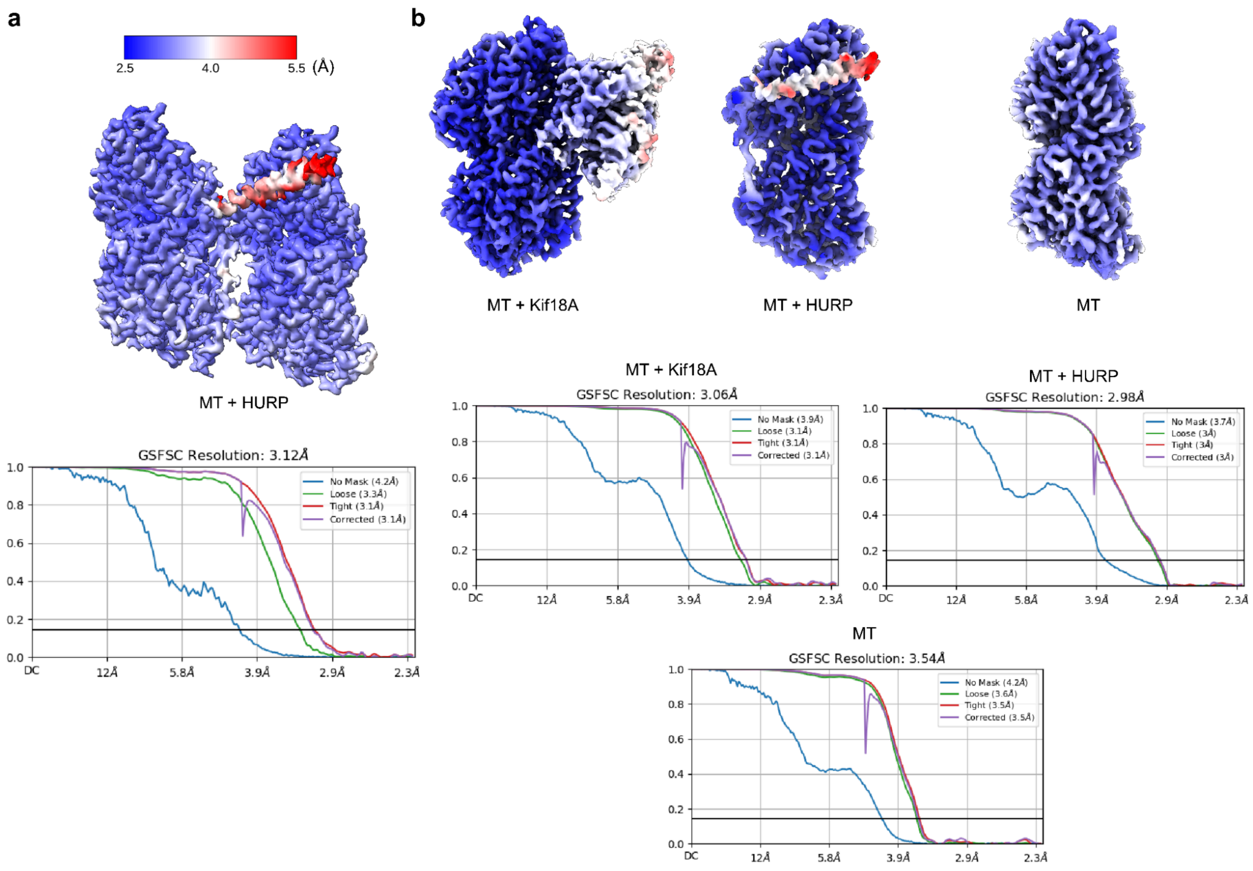

Supplementary Figure 7. Local resolution maps and FSC curves. a. Local resolution map (top) and Fourier Shell Correlation (FSC) plots (bottom) for microtubule-bound HURP. **b.** Local resolution maps (top) and Fourier Shell Correlation (FSC) plots (bottom) for the three classes produced after 3D classification of the microtubule-HURP-Kif18A$^{1\text{-}373}$ dataset.

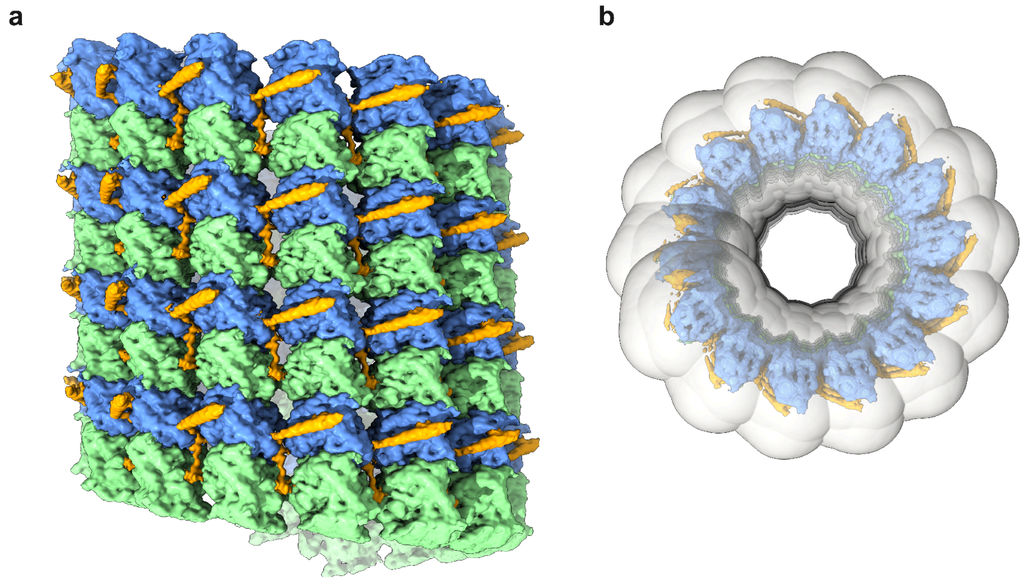

Supplementary Figure 8. Density for the HURP MTBD2 is absent in the cryo-EM reconstruction. a. Side view of an unsharpened microtubule-HURP$^{1\text{-}285}$ cryo-EM map (3.5 Å) visualized at low threshold (0.116) and without dust hiding. α-tubulin, β-tubulin and HURP MTBD1 are represented in green, blue and orange, respectively. **b.** Top view of the map in **a** showing the dilated mask used for refinement of particle poses in transparent gray.

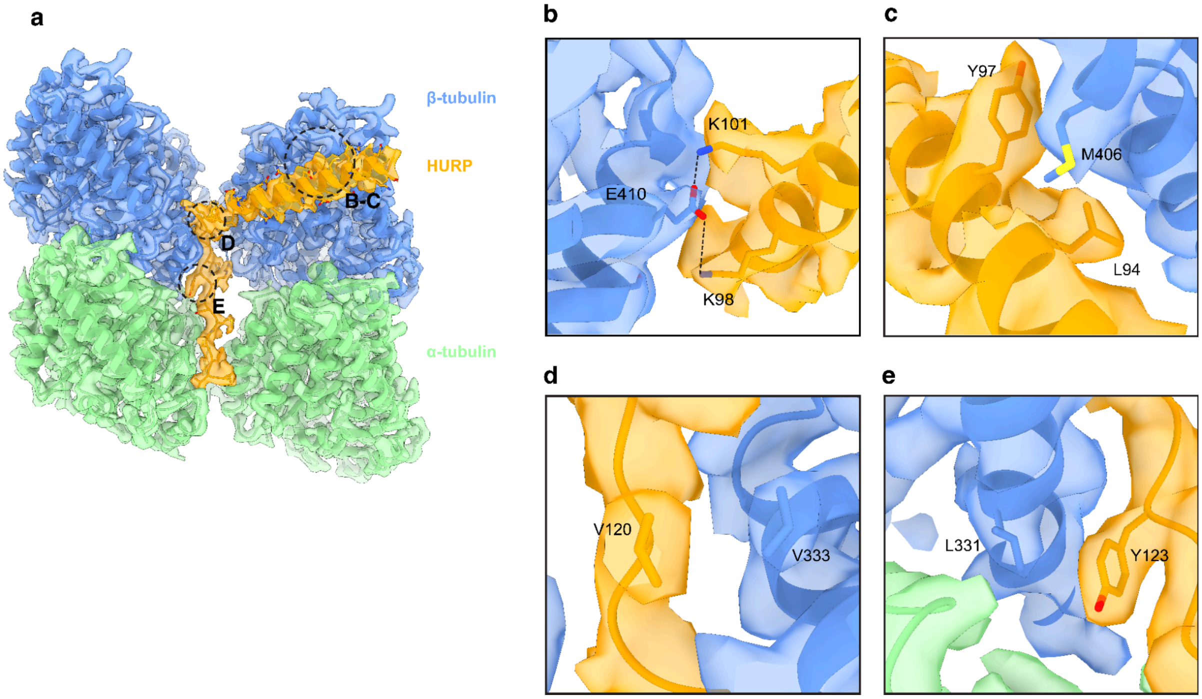

Supplementary Figure 9. Detailed interactions between HURP and tubulin. a. Final microtubule-HURP cryo-EM map. A single HURP molecule is shown for clarity. The refined model is overlaid on the map. Dashed circles indicate the regions shown in panels **b-e**. **b-e.** Additional interactions between HURP and tubulin.

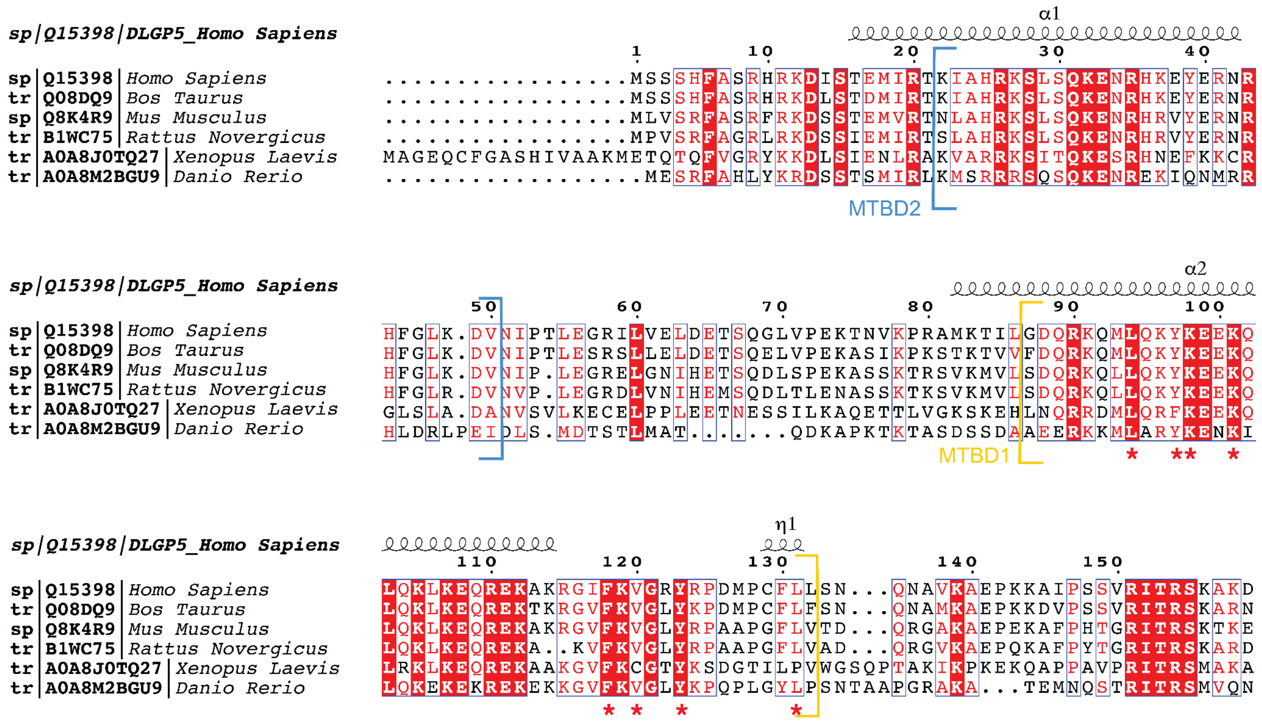

Supplementary Figure 10. HURP residues that interact with tubulin are conserved across species. Sequence alignment and conservation analysis for HURP from different species. The human version was set as a reference and residues that are seen to interact with tubulin in our cryo-EM structure are marked with red asterisks. ESPript3[1] was used to generate the figure, and secondary structure was annotated based on the AlphaFold[2] prediction for human HURP (Uniprot Q15398). Regions corresponding to MTBD2 (blue) and the structurally resolved part of MTBD1 (yellow) are indicated.

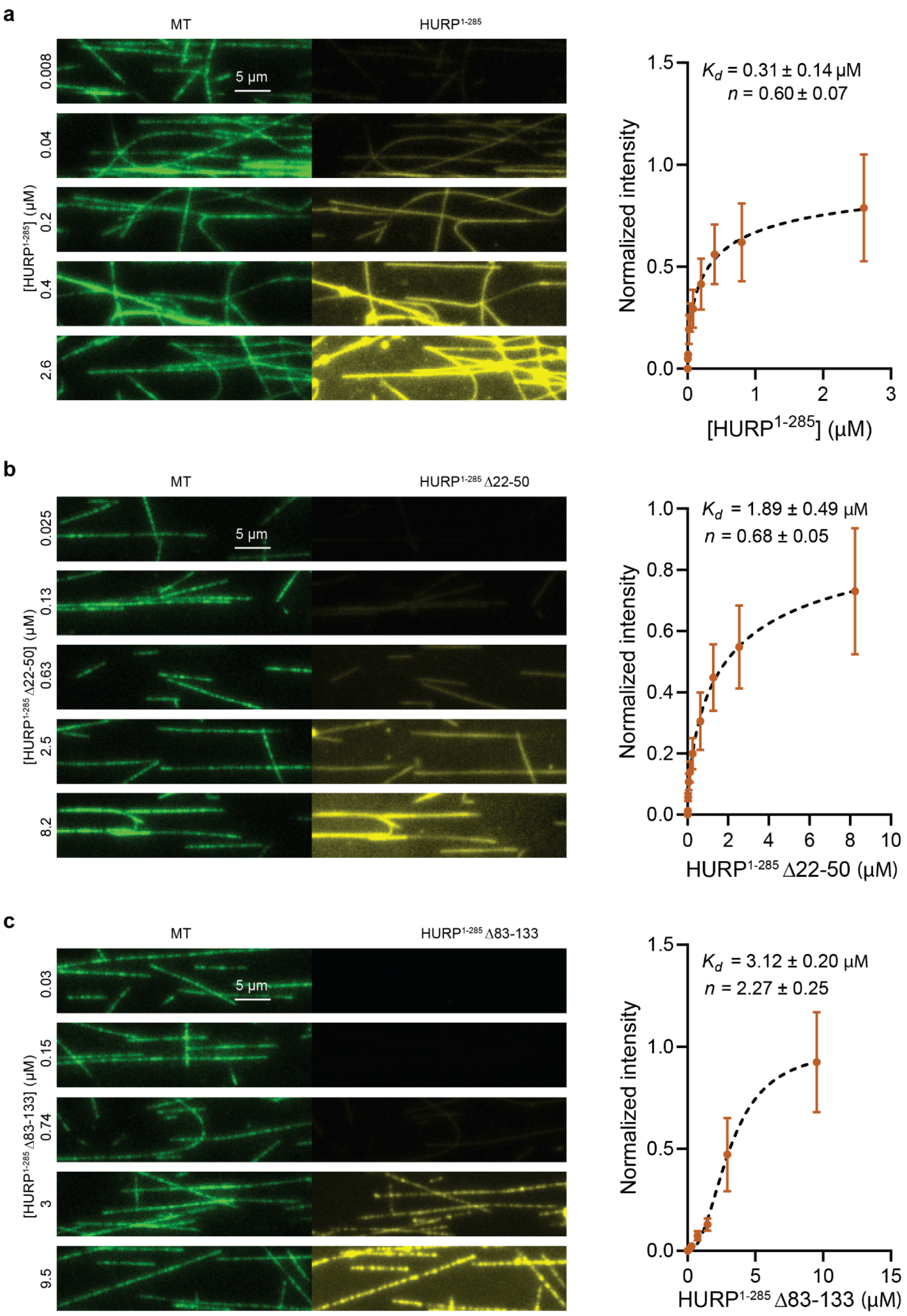

Supplementary Figure 11. HURP MTBD deletions impact microtubule binding. a. (Left) Representative images showing $HURP^{1-285}$ binding to microtubules at different concentrations. (Right) Quantification of $HURP^{1-285}$ binding to microtubules. **b.** (Left) Representative images

showing HURP$^{1-285}$Δ22-50 binding to microtubules at different concentrations. (Right) Quantification of HURP$^{1-285}$Δ22-50 binding to microtubules. **c.** (Left) Representative images showing HURP$^{1-285}$Δ83-133 binding to microtubules at different concentrations. (Right) Quantification of HURP$^{1-285}$Δ83-133 binding to microtubules. For **a**, **b** and **c**, The center circle and whiskers represent the mean and S.D., respectively. K_d is determined from a fit to a Hill equation (dashed curve, N = 20 microtubules for each condition). Source data are provided as a Source Data file.

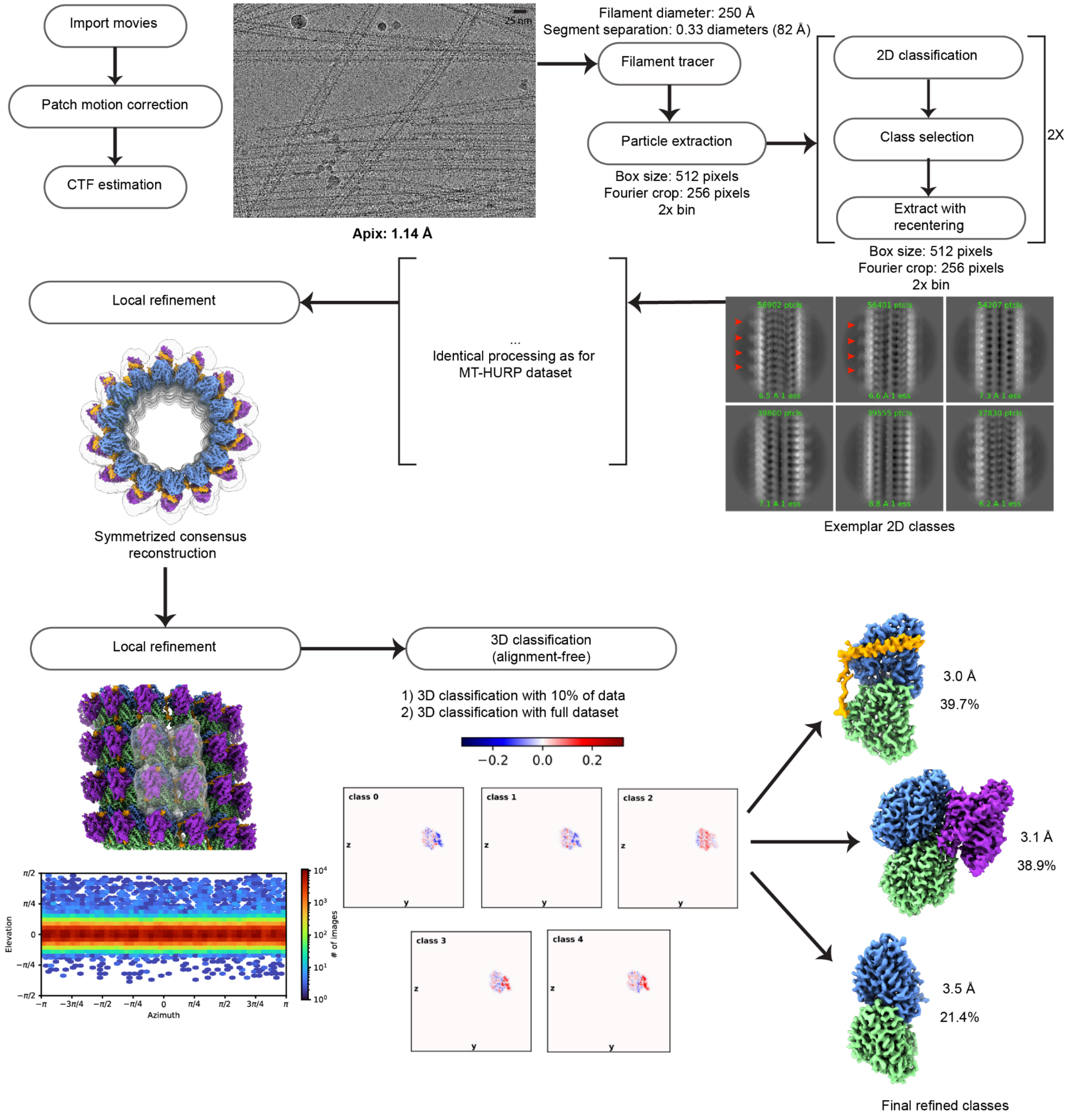

Supplementary Figure 12. Cryo-EM data processing for the microtubule-HURP-Kif18A dataset. Processing pipeline applied to the microtubule-HURP-Kif18A$^{1\text{-}373}$ dataset. Unless specified otherwise, all steps were performed in CryoSparc. Red arrowheads point to Kif18A density in the 2D class averages. In the 3D classification step, the blue-red key represents density variation in each class with respect to the input consensus reconstruction, with regions in blue and red containing significantly less or more density than the consensus, respectively. Masks are shown with transparent surfaces.

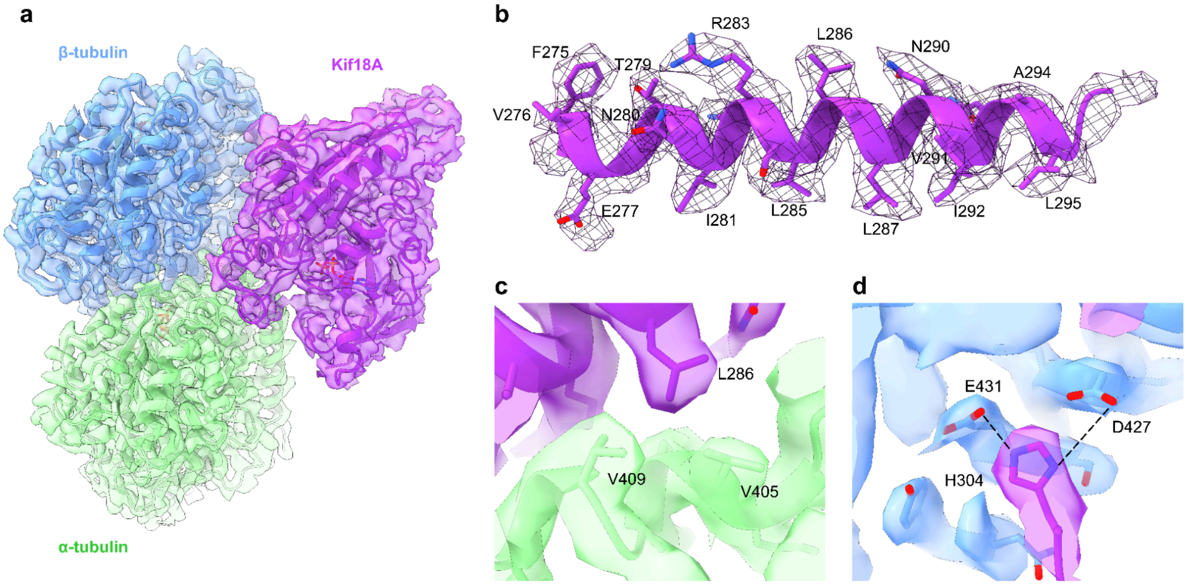

Supplementary Figure 13. Map quality and detailed interactions for the Kif18A-tubulin class. a. Surface representation of the cryo-EM density corresponding to the microtubule-Kif18A class, with a refined tubulin-Kif18A model fitted inside the map. α-tubulin, β-tubulin and Kif18A are represented in green, blue and purple, respectively. **b.** Isolated density in mesh representation for Kif18A's α4 helix, with the corresponding segment of the real-space refined atomic model of Kif18A. **c-d.** Selected detailed interactions between Kif18A and tubulin.

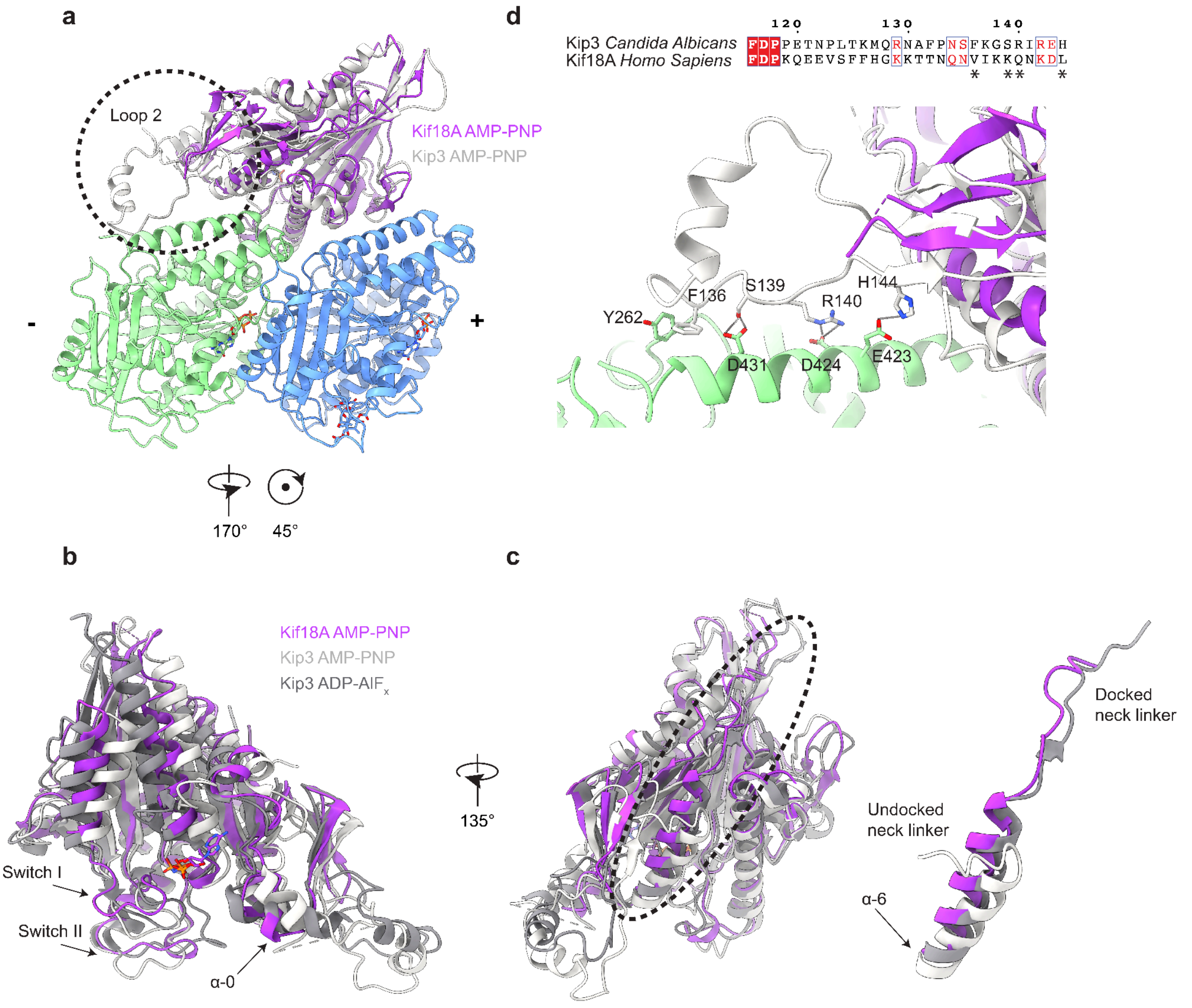

Supplementary Figure 14. Comparison between microtubule-bound human Kif18A and yeast Kip3 structures. a. Ribbon diagrams of the motor domains of human Kif18A (purple) and *C. Albicans* Kip3 (light gray, PDB 7TQX) in an ATP-mimicking state (AMP-PNP). α and β-tubulin are shown in green and blue, respectively, with the minus and plus-ends marked for reference. The two models were aligned to the β-tubulin subunit for comparison. A circle with dashed-lines marks Kip3 loop 2 and the surrounding structural elements. **b.** Ribbon diagrams of the motor domains of human Kif18A in an AMP-PNP state (purple), *C. Albicans* Kip3 in an AMP-PNP state (light grey, PDB 7TQX) and *C. Albicans* Kip3 in an ADP-AlF$_x$ state (dark grey, PDB 7TQY). The models were aligned to the β-tubulin subunit (not shown) for comparison. Structural elements involved in nucleotide binding pocket closure are marked with arrows. **c.** Rotated view of the models in **b.** with the neck linker area encircled by dashed-lines (left) and a detailed view of this region (right) showing the docked neck linkers for Kif18A (AMP-PNP) and Kip3 (ADP-AlF$_x$) and an undocked neck linker for Kip3 (AMP-PNP). **d.** (Top) Kif18A and Kip3 sequence alignment of loop 2 using ESPript3[1]. Kip3 residues that establish contacts with α-tubulin are marked with an asterisk. (Bottom) Magnified view of the circled area in **a**. Contacts between Kip3 loop 2 and α-tubulin are shown in atom representation.

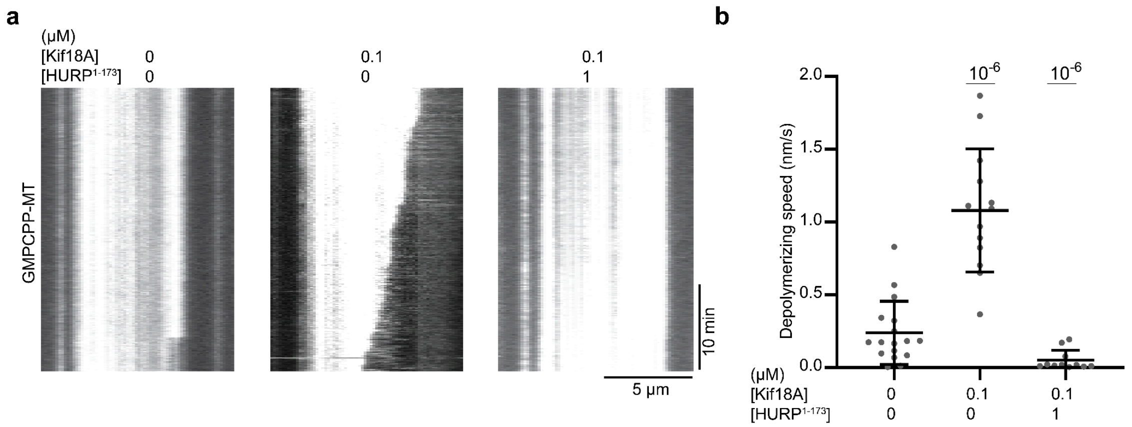

Supplementary Figure 15. Kif18A can depolymerize GMPCPP microtubules. a. Representative kymographs of GMPCPP-microtubule depolymerization with 0.1 µM Kif18A or 0.1 µM Kif18A + 1 µM HURP$^{1-173}$. **b**. Microtubule depolymerization speed with 0.1 µM Kif18A or 0.1 µM Kif18A + 1 µM HURP$^{1-173}$. (From left to right, N= 17, 13 and 11 kymographs). The center line and whiskers represent the mean and S.D., respectively. P values are calculated from a two-tailed t-test. Source data are provided as a Source Data file.

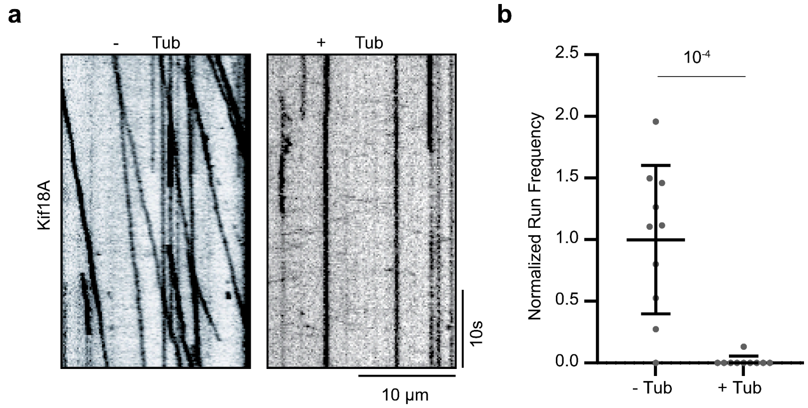

Supplementary Figure 16. Kif18A motility is affected by the presence of free tubulin. a. Representative kymographs showing the motility of full-length Kif18A with or without 2 mg/mL free tubulin. **b**. Normalized run frequency of full-length Kif18A with or without 2 mg/mL free tubulin (N = 10 kymographs for each condition). The center line and whiskers represent the mean and S.D., respectively. P values are calculated from a two-tailed t-test. Source data are provided as a Source Data file.

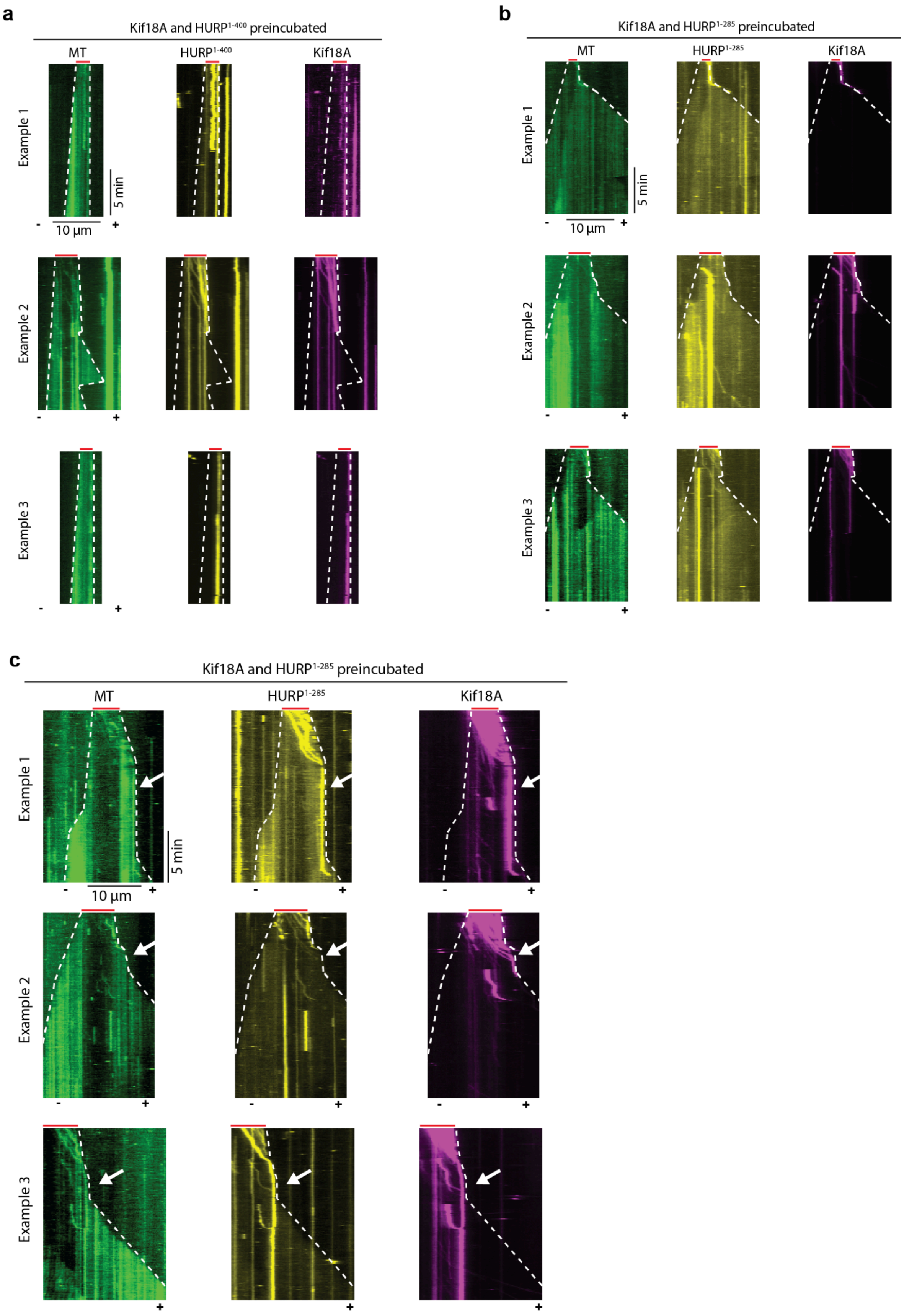
a
Kif18A and HURP1-400 preincubated
MT
HURP1-400
Kif18A
Example 1
Example 2
Example 3
5 min
10 µm
-
+
b
Kif18A and HURP1-285 preincubated
MT
HURP1-285
Kif18A
Example 1
Example 2
Example 3
5 min
10 µm
c
Kif18A and HURP1-285 preincubated
MT
HURP1-285
Kif18A
Example 1
Example 2
Example 3
5 min
10 µm

Supplementary Figure 17. Kif18A and HURP jointly control microtubule length. a-b. Additional examples of kymographs showing **(a)** Kif18A and HURP$^{1\text{-}400}$ or **(b)** Kif18A and HURP$^{1\text{-}285}$ collectively maintaining a constant microtubule length. **c.** Kif18A and HURP$^{1\text{-}285}$ collectively cap microtubules following initial plus-end growth. White dashed lines show the track of microtubule ends and red lines mark the position of the seeds.

Name	Microtubule - HURP$^{1\text{-}285}$	Microtubule - HURP$^{1\text{-}285}$ - Kif18A$^{1\text{-}373}$ (HURP class)	Microtubule - HURP$^{1\text{-}285}$ - Kif18A$^{1\text{-}373}$ (Kif18A class)	Microtubule - HURP$^{1\text{-}285}$ - Kif18A$^{1\text{-}373}$ (Tubulin class)
EMDB ID	XXX	XXX	XXX	XXX
Microscope	Arctica	Arctica	Arctica	Arctica
Voltage (kV)	200	200	200	200
Camera	K3	K3	K3	K3
Defocus range (μm)	0.8-2	0.8-2	0.8-2	0.8-2
Automation software	SerialEM	SerialEM	SerialEM	SerialEM
Frames	50	50	50	50
Total dose (electrons/Å²)	50	50	50	50
Pixel size (Å/pixel)	1.14	1.14	1.14	1.14
Number of micrographs	796	2611	2611	2611
Starting number of particles (pre-symmetry expansion)	99,992	1,151,884	1,151,884	1,151,884
Number of particles in final map (post-symmetry expansion)	353,980	2,405,078	2,349,581	1,296,716
Map sharpening B factor (Å)	-60	-100	-60	-80
Map sharpening methods	CryoSparc	CryoSparc	CryoSparc	CryoSparc
Symmetry	C1	C1	C1	C1
Overall resolution (Å)	3.1	3.0	3.1	3.5

Resolution range of map (min-75th percentile) (Å)	2.8-4.1	2.5-5.6	2.6-4.0	2.9-4.4

Metric	Value	
	Microtubule - HURP	Microtubule - Kif18A
Initial model used (PDB ID)	6DPV	5OCU
Refinement package	Phenix	Phenix
C-beta outliers (%)	0	0
Rotamer outliers (%)	0.20	0
All-atom Clash score	9.85	8.12
MolProbity score	1.62	1.81
Ramachandran plot (outliers/favored) (%)	0.00/97.49	0.17/94.65
Ligand	6	5
Protein residues	1768	1175
R.m.s.d. of bond lengths (Å)	0.01	0.004
R.m.s.d. of bond angles (°)	1.058	0.632
CC (mask)	0.84	0.81
CC (volume)	0.83	0.74

Supplementary Table 1. Cryo-EM data collection parameters and model refinement statistics.

Supplementary References

1. Robert, X. & Gouet, P. Deciphering key features in protein structures with the new ENDscript server. *Nucleic Acids Res.* **42**, W320–W324 (2014).
2. Jumper, J. *et al.* Highly accurate protein structure prediction with AlphaFold. *Nature* **596**, 583–589 (2021).
